# The Typical Presentation Spectrum of Deep Vein Thrombosis Associated with Inferior Vena Cava Malformations

**DOI:** 10.1155/2016/4965458

**Published:** 2016-07-10

**Authors:** Agostino Pozzi, Mustapha A. El Lakis, Jad Chamieh, Beatriz Barberà Carbonell, Fabio Villa

**Affiliations:** ^1^San Raffaele Hospital, Vita-Salute San Raffaele University, 20132 Milan, Italy; ^2^Department of Thoracic Surgery, Virginia Mason Medical Center, Seattle, WA 98101, USA; ^3^Department of Surgery, Washington University in St. Louis, St. Louis, MO 63110, USA; ^4^Ente Ospedaliero Cantonale, 6500 Lugano, Switzerland; ^5^Ente Ospedaliero Cantonale, 6900 Bellinzona, Switzerland

## Abstract

Congenital malformations of the inferior vena cava (IVC) are rare and underreported. They can be a risk factor for deep venous thrombosis (DVT) as a result of inadequate venous drainage of the lower extremities through collateral circulation. The significant number of cases reported in the literature highlights their importance, warranting investigating their existence in younger individuals with idiopathic DVT of the lower extremities and pelvic veins. In this systematic review, we depict the typical presentation of IVC malformations, their management, and the management of their associated DVT.

## 1. Introduction 

The inferior vena cava (IVC) congenital variants include agenesis, interruption with azygous or hemiazygous continuation, web formation, hypoplasia, left-sided location, and duplication; these conditions may be identified solely or in conjunction with one another. Their projected prevalence in the general population is approximately 4% [1]; IVC anomalies are usually asymptomatic and are incidentally diagnosed during investigations for other medical conditions. Different theories attempted explaining their unclear etiologies such as aberrant embryologic development of the posterior cardinal, subcardinal, supracardinal, and vitelline veins at around the sixth gestational week [2] or the development of IVC thrombosis during the intrauterine or perinatal life [3]. The most reported anatomic anomaly in this case series is IVC agenesis ranging from 1/100 to 1/200,000 in the general population [4] and in 5% of deep venous thrombosis (DVT) patients younger than 30 years [5]. Evidence has shown that patients with agenesis of inferior vena cava (AIVC) are prone to develop deep vein thrombosis (DVT) of the lower extremities at a younger age [6]. This warrants investigating IVC malformation as an etiologic factor in young patients diagnosed with idiopathic DVT.

Until recently and due to the rarity of this condition, only single case reports described DVT in patients with IVC malformations and thereby its clinical presentation, management, and sequelae remain poorly understood. This paper attempts to report all cases of DVT in patients with IVC anomalies in the literature along with a review of symptomatology, diagnosis, and treatment. We aim to raise awareness of IVC anomalies as a risk factor in young patients with idiopathic DVT.

## 2. Methods

We conducted a systematic search on PubMed, Medline, Ovid, Google Scholar, and Cochrane data search engines of English language case reports and case series reporting DVT in patients with agenesis, hypoplasia, and any other malformations of the inferior vena cava. The search was performed by three authors independently. Eighty-six publications have been identified, predominantly case reports from 1988 to 2015, totaling 188 patients. Four publications were excluded because of lack of sufficient and relevant data, given that our goal is addressing several valuable questions in clinical practice. We focused our statistical analysis on the demographic data of the patients with IVC anomalies, clinical DVT presentation, comorbidities, contribution of thrombophilia screening, and therapeutic management.

## 3. Results

We identified 188 patients with IVC malformation presenting with DVT. Patient characteristics are presented in Table 1. Mean age at diagnosis of DVT was 27.5 ± 11.4 years (min. 9, max. 72) particularly; 138 patients (73.4%) were under 30 years of age. Male to female ratio was 4 : 1.

Patients typically presented with leg swelling, leg pain, lower back pain, and/or abdominal pain. Only four patients were asymptomatic, and one patient was admitted for polytrauma with a subsequent diagnosis of DVT.

In the majority of cases, the diagnostic workup of DVT and IVC anomalies consisted of ultrasonography (US) followed by computed tomography (CT) scanning with intravenous contrast (25%). Other modalities such as magnetic resonance imaging (MRI) and venography in combination with US and CT were occasionally performed.

Imaging reported bilateral DVT in 48.9% of the cases and similar prevalence of right-sided only (25.3%) or left-sided only (25.8%) DVT (Table 1). All patients were diagnosed with one of the following IVC anomalies: prerenal IVC agenesis (13.8%), infrarenal IVC agenesis (17%), postrenal IVC agenesis (0.5%), infrahepatic IVC agenesis (7%), IVC hypoplasia (4.2%), IVC duplication (2.7%), and IVC agenesis not further classified (54.8%). In 15 cases, associated anomalies were also present, largely right kidney aplasia (7 patients) and left kidney aplasia (5 patients). The others were polysplenia (2 patients) and right hepatic lobe agenesis (1 patient).

After initial imaging, 168 patients (90%) were screened for genetic blood coagulation disorders with positive findings in 68 (40.5%). The most prevalent was factor V Leiden mutation in 19 patients followed by prothrombin G20210A mutation (8 cases), protein C or protein S deficiency (4 cases), and lupus anticoagulant (4 cases). The others had one of the following: antiphospholipid antigens, hyperhomocysteinemia, factor VIII elevation, and antithrombin III deficiency. Twenty-four patients were found positive for two or three thrombophilic factors (Table 2).

Management mostly consisted of anticoagulation with unfractionated heparin (35.2%) or low molecular weight heparin (LMWH) (22.1%). DVT management in 35 patients required one of the following surgical procedures: prosthetic bypass (11.7%), pharmacomechanical catheter-directed thrombolysis (PCDT) (8.4%), surgical thrombectomy (1.9%), or IVC Greenfield filter (0.6%) as summarized in Table 3. Almost all patients (99%) were discharged on vitamin K antagonists (VKA), mainly warfarin, and/or elastic compression stockings for a period of at least 6 months. Factor Xa inhibitors (Rivaroxaban and Apixaban) were prescribed in two cases.

Sixty cases were followed up with imaging studies, mainly duplex venous ultrasound, for a mean period of 12.9 ± 12.4 months [range: 30 days–5 years]. Recurrence was demonstrated in 23 and resolution in 37 patients.

## 4. Discussion

A thrombophilic state leading to venous thrombosis can be inherited or acquired. The most prevalent inherited hypercoagulable states are factor V Leiden mutation, prothrombin gene mutation, MTHFR gene mutation, lupus anticoagulant, defects in protein S, protein C, and antithrombin, and dysfibrinogenemia. The most frequently encountered acquired risk factors are immobility for more than 48 hours in the past month, surgery, malignancy, infection in the past three months, current hospitalization, and pregnancy. A number of drugs have also been associated with an increased risk of venous thrombosis: oral and transdermal contraceptives, hormone replacement therapy, glucocorticoids, and others. Inferior vena cava malformations, especially if concomitantly present with other thrombophilic conditions, are a potential risk factor for DVT as they might result in insufficient venous drainage of the lower extremities through collateral circulation resulting in blood stasis.

### 4.1. Incidence

The estimated prevalence of congenital IVC malformations is difficult to determine but it has been projected to be approximately 4% in the general population [1]. They usually remain asymptomatic during childhood and manifest themselves in the early adulthood, especially in the presence of other thrombotic risk factors [7]. The rate of DVT in patients younger than 30 years of age with IVC malformations is estimated to be 5% versus 0.5–0.6% in the same group with no IVC anomalies [8]; moreover, the incidence of bilateral thrombosis in patients with IVC anomalies ranges from 35.4% to 60% [9, 10].

Literature has demonstrated that 90% of IVC anomalies involve the suprarenal segment and only 6% involve the renal or infrarenal segments, making absent infrarenal IVC the rarest congenital anomaly [11, 12]. In contrast, in our case series, infrarenal IVC agenesis was the most prevalent among the IVC anomalies (17%), followed by prerenal and infrahepatic IVC, and it is important to note that IVC agenesis was not further classified in 54.8% of the cases.

### 4.2. Coagulation Profile

Screening for thrombophilia is important when evaluating DVT patients having an IVC anomaly as it would affect the management approach in terms of duration of anticoagulation, prevention, and follow-up strategies. Thrombophilia screening includes the assessment of factor V Leiden mutation, prothrombin G20210A mutation, antiphospholipid antibodies, methylenetetrahydrofolate reductase gene mutation, lupus anticoagulant, antithrombin, and proteins C and S activities [13]. Rogers et al. reported that 44% of patients with absent IVC also had a positive thrombophilia screening [14]. We report a similar incidence rate in this series (40.5%) suggesting a complementary causal relation between IVC anomalies and thrombophilia on the one hand and pathogenesis of DVT on the other hand.

### 4.3. Symptoms

Low-back and abdominal pain have been reported as the most common initial presenting symptoms [15], favoring the hypothesis that the thrombotic process initially involves the IVC and only afterwards descends to the pelvic and lower extremities veins. In our series, back and abdominal pain, with an incidence of 17% and 12.8%, respectively, always manifested themselves together with DVT symptoms such as lower extremity pain and edema.

### 4.4. Diagnosis

Ultrasonography of the lower extremities is the first imaging test to be performed when DVT is suspected. The IVC is not typically investigated for anatomic anomalies by US as its accuracy is limited for being operator-dependent and secondary to interference of bowel gas and body habitus in overweight and obese patients [1]. In this case series, 83% of patients underwent US as the first imaging modality but the most common diagnostic tool was CT scan with intravenous contrast (86%). Abdominal CT scan with IV contrast shows enhancement in the renal and suprarenal IVC but may also show admixture artifacts in the infrarenal portion of the IVC; however, delaying the scan from 60–70 to 70–90 seconds after contrast administration allows a more uniform enhancement of the entire IVC [16]. Despite its advantage of being a rapid noninvasive imaging modality, MRI imaging is now replacing CT as the optimal investigative tool avoiding radiation and giving more accurate delineation of thrombus as well as any IVC anomaly. MRI is also used to follow up patients to determine morphological changes in the thrombus following therapy [17]. Only 56% of patients in our case series underwent MRI; however, in the case reports from 2014 onwards, 79% underwent an MRI underlining the tendency mentioned above. Moreover, venography and other invasive diagnostic tools are being replaced by US, CT, and MRI.

### 4.5. Management, Sequelae, and Follow-Up

Controversy exists regarding the evidence-based management approach of DVT associated with IVC abnormalities. Given the rarity of this condition, performing clinical trials to determine the best treatment strategy is prohibitive. From this case series, the largest on the topic, we try to derive a trend in both the inpatient acute setting and the outpatient management.

As the diagnosis of DVT associated with IVC anomalies is confirmed, patients are treated either conservatively with unfractionated heparin and LMWH (35.2% and 22.1% in our case series) or surgically for severe venous insufficiency not correctable with anticoagulation alone manifested by nonhealing lower extremity ulcers. Surgical options were prosthetic bypass (11.7%), pharmacomechanical catheter-directed thrombolysis (PCDT) (8.4%), surgical thrombectomy (1.9%), or IVC Greenfield filter (0.6%). PCDT, compared to systemic anticoagulation alone, significantly decreases the thrombus burden and incidence of recurrent DVT in patients with extensive iliofemoral DVT [18]. PCDT could be a treatment option in patients with symptomatic iliofemoral DVT, good functional status, life expectancy of 1 year or more, and low risk of bleeding [19]. However, studies focusing on the long-term outcomes following PPCDT are desperately needed as evidence is lacking.

In the outpatient setting, adjustment of modifiable risk factors (i.e., oral contraceptive pills, immobilization, and major physical activity), compression stockings, and long-term anticoagulation are the treatment of choice. In this case series, patients were prescribed oral vitamin K antagonists, mostly warfarin, with a target international normalized ratio (INR) range of 2-3. Factor Xa inhibitors (Rivaroxaban and Apixaban) were prescribed in two cases. 17% of patients were prescribed compression stockings. There is no consensus concerning the duration of anticoagulation. In this case series, 11 patients discontinued oral anticoagulation therapy at 6 months and 8 patients at 1 year. All others were on long-term anticoagulation that was decided on case-by-case basis according to treating physicians with or without compression stockings. At least 3 to 6 months of anticoagulation is required for DVT associated with IVC agenesis. While the majority of patients might have other risk factors, prolonged oral anticoagulation and compression stockings are recommended [20]. Follow-up, mainly by US, aims at detecting DVT recurrence. Currently, no data exists on long-term morbidity and mortality following DVT associated with IVC abnormalities.

## 5. Conclusion

A high index of suspicion for inferior vena cava anomalies should be considered in young patients presenting with deep vein thrombosis. We suggest being liberal in screening for IVC anomalies in this high risk group.

## Figures and Tables

**Table 1:** Characteristics of the patients.

Characteristics		
*Number of patients*	188	

*Sex: male/female *	142/46	

*Age at diagnosis of DVT *		
Mean (years ± SD)	27.5 ± 11.4	
Range (min.–max.)	9–72	

*DVT site n, (%)* ^*∗*^		
Left side	47	(25.8%)
Right side	46	(25.3%)
Bilateral	89	(48.9%)

*Type of IVC malformation n, (%)*		
Infrarenal IVC agenesis	32	(17%)
Prerenal IVC agenesis	26	(13.8%)
Postrenal IVC agenesis	1	(0.5%)
Infrahepatic IVC agenesis	13	(7%)
IVC hypoplasia	8	(4.2%)
IVC duplication	5	(2.7%)
Nonspecified IVC agenesis	103	(54.8%)

DVT, deep vein thrombosis; IVC, inferior vena cava; SD, standard deviation.

^*∗*^Out of 182, 6 cases are not documented.

**Table 2:** Prevalence of hereditary disorders of coagulation.

Coagulation defect (*n*, %)		
Factor V Leiden	19	(10.1%)
Prothrombin G20210A mutation	8	(4.3%)
Protein S/C deficiency	4	(2.1%)
Lupus anticoagulant	4	(2.1%)
Antiphospholipid antibodies	3	(1.6%)
Hyperhomocysteinemia	2	(1.1%)
Factor VIII elevation	2	(1.1%)
Antithrombin III deficiency	2	(1.1%)
Two thrombophilic factors positive	17	(9.1%)
Three thrombophilic factors positive	7	(3.7%)
Negative thrombophilia test	100	(53.1%)
Not tested	20	(10.6%)

**Table 3:** Hospital and long-term treatment of DVT and follow-up.

*Hospital management n*, (%)^*∗*^		
Unfractionated heparin	54	(35.2%)
LMWH	34	(22.1%)
Prosthetic bypass	18	(11.7%)
PCDT	13	(8.4%)
Surgical thrombectomy	3	(1.9%)
IVC-filter	1	(0.6%)
Nonspecified anticoagulation	31	(20.1%)

*Long-term treatment n, (%)*		
Prolonged/lifelong VKA	135	(71.8%)
Prolonged/lifelong VKA +	32	(17%)
Compression stockings		
6-month VKA	11	(5.8%)
12-month VKA	8	(4.4%)

*Follow-up duration in months* ^*∗∗*^		
Mean ± SD	12.85 ± 12.4	
Range	1–60	

DVT, deep vein thrombosis; IV, intravenous; LMWH, low molecular weight heparin; PCDT, pharmacomechanical catheter-directed thrombolysis; VKA, vitamin K antagonist; SD, standard deviation.

^*∗*^Out of 154, 34 cases are not reported.

^*∗∗*^Reported only in 60 cases.
